# Correction: High-throughput screening of FDA-approved drugs identifies colchicine as a potential therapeutic agent for atypical teratoid/rhabdoid tumors (AT/RTs)

**DOI:** 10.1039/d5ra90136g

**Published:** 2025-11-18

**Authors:** Phongthon Kanjanasirirat, Kedchin Jearawuttanakul, Sawinee Seemakhan, Suparerk Borwornpinyo, Patompon Wongtrakoongate, Suradej Hongeng, Sitthivut Charoensutthivarakul

**Affiliations:** a School of Bioinnovation and Bio-based Product Intelligence, Faculty of Science, Mahidol University Bangkok 10400 Thailand sitthivut.cha@mahidol.ac.th +66-2-201-5899; b Department of Pathobiology, Faculty of Science, Mahidol University Bangkok 10400 Thailand; c Excellent Center for Drug Discovery (ECDD), Faculty of Science, Mahidol University Bangkok 10400 Thailand; d Department of Biotechnology, Faculty of Science, Mahidol University Bangkok 10400 Thailand; e Department of Biochemistry, Faculty of Science, Mahidol University Bangkok 10400 Thailand; f Center for Neuroscience, Faculty of Science, Mahidol University Bangkok 10400 Thailand; g Department of Pediatrics, Faculty of Medicine Ramathibodi Hospital, Mahidol University Bangkok 10400 Thailand

## Abstract

Correction for ‘High-throughput screening of FDA-approved drugs identifies colchicine as a potential therapeutic agent for atypical teratoid/rhabdoid tumors (AT/RTs)’ by Phongthon Kanjanasirirat *et al.*, *RSC Adv.*, 2025, **15**, 12331–12341, https://doi.org/10.1039/D5RA01341K.

The authors regret the omission of Tanawadee Khumpanied from the Acknowledgements. The correct Acknowledgements are as shown here.

This research was partially supported by the Ramathibodi Foundation and Thailand Center of Excellence for Life Sciences (TCELS), and also by the Faculty of Science, Mahidol University *via* the Reinventing University Project. The authors would like to thank Dr Witchuda Saengsawang, Dr Jiraporn Panmanee and Dr Sujira Mukda for discussions and Ms Tanawadee Khumpanied for preliminary data collection.

The Royal Society of Chemistry apologises for these errors and any consequent inconvenience to authors and readers.

